# Multifactorial stratified analysis of two novel metabolism-related indices for predicting stroke in mild cognitive impairment

**DOI:** 10.3389/fneur.2026.1814327

**Published:** 2026-07-13

**Authors:** Boyuan Qiu, Hongliang Tang, Liangyuan Tan, Peng Yang, Wenhui Wang, Peipei Yang, Kailong Wang

**Affiliations:** 1 Guangxi University of Chinese Medicine, Nanning, Guangxi, China; 2 The First Affiliated Hospital of Guangxi University of Chinese Medicine, Nanning, Guangxi, China

**Keywords:** body roundness index, CHARLS, C-reactive protein-triglyceride glucose index, mild cognitive impairment, stroke

## Abstract

**Objectives:**

This study aimed to evaluate the predictive value of two novel metabo-lism-related indices, the C-reactive protein-triglyceride glucose index (CTI) and the Body Roundness Index (BRI), for new-onset stroke in patients with mild cognitive impairment (MCI). A systematic stratified analysis based on various factors was con-ducted to refine clinical strategies for the primary prevention of stroke.

**Methods:**

Data were obtained from the China Health and Retirement Longitudinal Study (CHARLS). A total of 907 eligible participants were included in this cohort study. Multivariable logistic regression models were employed to assess the associations of CTI and BRI with stroke risk. Exploratory stratified analyses were performed using piecewise linear regression and receiver operating characteristic (ROC) curves based on key behavioral and demographic characteristics, with optimal cutoff values calculated for each stratum. Subgroup analyses were conducted to verify the stability of the findings.

**Results:**

During the follow-up period, 81 participants (8.93%) experienced a stroke. After adjusting for potential confounding factors, each one-unit increase in CTI was associated with a 34% higher risk of stroke among MCI patients (Odds Ratio (OR): 1.34, 95% Confidence Interval (CI): 1.04–1.72, *p* < 0.05), demonstrating a significant increasing trend (*P* for trend < 0.05). In contrast, BRI showed no significant correlation with stroke occurrence (OR: 1.12, 95% CI: 0.97–1.30, *p* > 0.05). The multifactorial stratified analysis revealed that among MCI patients who smoked, within the CTI range below 9.91, each unit increase in CTI was significantly associated with an elevated stroke risk (OR: 1.81, 95% CI: 1.09–3.01, *p* < 0.05). The predictive performance of CTI demonstrated no significant heterogeneity across populations with different characteristics (Areas Under the Curve (AUCs) ranged from 0.55 to 0.61), and the ROC analysis identified optimal CTI cutoff values ranging from 8.20 to 9.28 across subgroups. Subgroup analyses further showed that the association between CTI and stroke was more pro-nounced in females, married individuals, and patients aged below 65 years (*p* < 0.05).

**Conclusion:**

Among middle-aged and elderly Chinese patients with MCI, CTI predicts stroke risk better than BRI.

## 1 Introduction

Stroke, as defined by the American Heart Association (AHA) and the American Stroke Association (ASA), involves acute central nervous system infarction or hemorrhage of vascular origin, leading to neurological impairments (1). Classified primarily into ischemic and hemorrhagic forms, ischemic stroke accounts for more than 80% of cases and constitutes a significant global health issue responsible for substantial adult morbidity and mortality (2–4). Mild cognitive impairment (MCI) is a transitional stage between normal aging and dementia, characterized by mild reductions in memory and other cognitive abilities that do not notably impair daily living (5). Patients with MCI are generally considered at high risk of developing Alzheimer’s disease (6). However, existing evidence indicates that MCI and stroke share multiple risk factors, suggesting common pathophysiological mechanisms (7, 8). A longitudinal study further indicated that MCI is an independent risk factor for subsequent stroke (9). Additionally, community-based studies among elderly populations in rural China confirmed a significant correlation between MCI and a history of stroke (10). These findings collectively demonstrate a close, bidirectional relationship between MCI and stroke. In this context, among patients with established MCI, identifying specific indicators capable of predicting subsequent stroke is of considerable clinical importance, given that this population already faces a compounded burden of neurocognitive and cerebrovascular risk.

Metabolic disorders have been established as independent risk factors for vascular diseases. Common metabolic conditions can specifically damage vascular structures, leading to vascular endothelial dysfunction, microthrombosis, and hemodynamic changes (11, 12). Moreover, metabolic disorders are significantly associated with neurodegenerative diseases (13). A recent global report from the AHA highlighted that 87% of stroke risk is attributable to modifiable factors, including obesity, hyperglycemia, and dyslipidemia (14). This evidence suggests that metabolic disorders may constitute a common pathological foundation linking metabolic impairment, cognitive decline, and cerebrovascular health. Therefore, a focus on metabolic parameters might enable more accurate identification of individuals with MCI who are at high risk for stroke.

Recently developed composite indicators integrating multiple physiological and pathological parameters offer improved diagnostic and prognostic capabilities compared to traditional single-factor measures. Insulin resistance and vascular inflammation are central contributors to both atherosclerosis and stroke pathogenesis (15–17). Notably, the triglyceride-glucose index (TyG) and C-reactive protein (CRP) serve as reliable indicators of insulin resistance and systemic inflammatory response, respectively (18, 19). In 2022, the C-reactive protein-triglyceride glucose index (CTI) was proposed, combining TyG and CRP to comprehensively reflect insulin resistance and chronic inflammation (20). The Body Roundness Index (BRI) quantifies visceral fat accumulation through a geometric model, demonstrating better predictive value for metabolic disease risk compared to the body mass index (21). Notably, the predictive value of an index may vary according to patients’ certain behavioral characteristics and basic demographic features. However, there remains a research gap in the comprehensive investigation of the predictive value of these two indices for stroke risk in the MCI population.

Accordingly, leveraging the China Health and Retirement Longitudinal Study (CHARLS) database, this study systematically evaluated the predictive efficacy of CTI and BRI across different subgroups of MCI patients through multifactorial stratified analysis. The findings are expected to provide a reference for implementing targeted interventions related to metabolic mechanisms and informing strategies for the primary prevention of stroke.

## 2 Methods

### 2.1 Study design

Data analyzed in the current investigation were sourced from CHARLS, a comprehensive, nationally representative, interdisciplinary longitudinal survey designed to gather detailed data from Chinese citizens aged 45 years and above. Employing a multistage sampling approach, CHARLS initiated its baseline survey in 2011, encompassing 17,708 individuals from 28 provinces across China. Follow-up evaluations have been consistently performed every 2–3 years (22). This study incorporated data spanning four waves of the CHARLS (2011, 2013, 2015, and 2018). The 2011 wave served as the baseline, providing demographic, lifestyle, and blood biochemistry data, including fasting plasma glucose, triglycerides, and C-reactive protein required for CTI calculation. As blood biochemistry assessments were only conducted in the 2011 and 2015 waves, all CTI-related variables were derived exclusively from the 2011 baseline data. Follow-up waves in 2013, 2015, and 2018 were used to identify new-onset stroke events during the follow-up period. Ethical clearance was obtained from the Institutional Ethics Committee of Peking University, and written informed consent was obtained from all individuals who participated.

### 2.2 Study population

**Figure 1:**
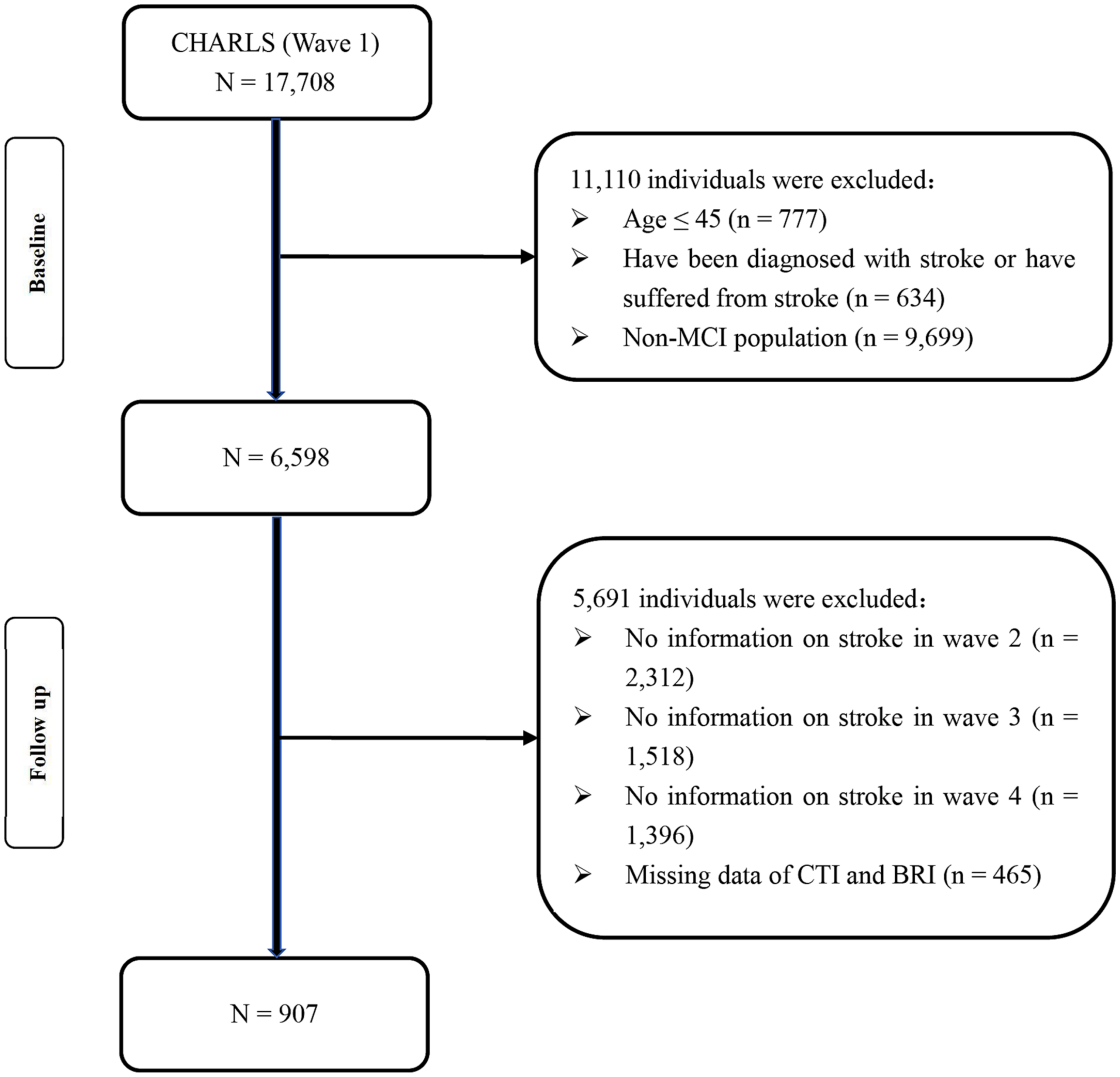
Flow diagram of participant selection.

Initially, 17,708 participants from the baseline survey were included. Ultimately, 907 participants remained after excluding 16,801 individuals based on the following criteria: age ≤45 years (*n* = 777), diagnosed with stroke at baseline (*n* = 634), absence of MCI (*n* = 9,699), unavailable CTI and BRI data (*n* = 465), and missing stroke follow-up information (*n* = 5,226). The detailed screening process is illustrated in Figure 1.

### 2.3 Measurements

#### 2.3.1 CTI

The CTI calculation involved assessing fasting plasma glucose (FPG), triglycerides (TG), and CRP. Specifically, CTI values were computed using the equation (20):

$\mathrm{CTI}=0.412\times \ln\;[\mathrm{CRP}\;(\mathrm{mg}/L)]+\ln\;[\begin{array}{l} \mathrm{TG}\;(\mathrm{mg}/\mathrm{dL})\times \\ \mathrm{FPG}\;(\mathrm{mg}/\mathrm{dL})/2 \end{array}].$

Following overnight fasting, venous blood samples were drawn by qualified technicians and subsequently processed at Capital Medical University’s central laboratory in China. These blood samples were collected during the 2011 baseline survey wave.

#### 2.3.2 BRI

BRI calculation required waist circumference (WC) and height measurements. Height was measured with participants standing barefoot on a flat surface. WC was measured at the midpoint between the lowest rib and the top of the iliac crest. The formula was (21):

$\mathrm{BRI}=364.2–365.5\times {\left[1-{\left(\mathrm{WC}/2\pi \right)}^{2}/{\left(0.5\times \text{Height}\right)}^{2}\right]}^{0.5}.$

#### 2.3.3 Cognitive function assessment

Cognitive function was assessed using the CHARLS cognitive module, comprising two components: the Word Recall Test, including immediate and delayed recall tasks (each scored out of 10 points, maximum 20 points total), and the Mental Status Test, including the Telephone Interview for Cognitive Status (maximum 10 points) and a graphic drawing task (1 point), yielding a maximum total cognitive score of 31 points. MCI was defined according to the aging-associated cognitive decline criteria: participants whose total cognitive score fell more than one standard deviation below the mean of their age group were classified as having MCI, with age stratified in five-year intervals. Detailed age-specific cutoff values and MCI prevalence by age group are provided in Supplementary Table 1 of reference (23).

#### 2.3.4 Stroke assessment

The primary endpoint of this investigation was the initial occurrence of stroke. Stroke cases were identified by trained assessors using a standardized question: “Have you ever been diagnosed with stroke by a physician?” (22). To ensure data accuracy, validation and verification were conducted by the study team.

#### 2.3.5 Covariates

Detailed information from participants was systematically collected through physical evaluations, structured questionnaires, and direct interviews. Variables regarding demographics incorporated educational background, marital condition, age, gender, and residential location, whereas lifestyle-related parameters included tobacco use and alcohol intake.

### 2.4 Statistical analysis

Continuous variables were expressed either as median and interquartile range (IQR) or as mean ± standard deviation (SD), depending on their distribution characteristics. Frequencies and proportions were used to present categorical data. Differences at baseline between participants who did or did not experience new-onset stroke were evaluated using t-tests and chi-square analyses. Multivariate logistic regression was applied to assess the independent associations between stroke risk and the indices CTI and BRI, producing odds ratios (OR) along with their 95% confidence intervals (CI). Additionally, trend analyses were performed by treating quartile classifications of CTI and BRI as continuous predictors in these logistic regression models. Further exploratory stratified analyses were conducted based on key behavioral and demographic characteristics. First, the potential threshold effects of the metabolic indices on stroke incidence were explored using piecewise linear regression combined with smoothing curves, stratified by drinking and smoking status. Subsequently, receiver operating characteristic (ROC) curve analysis was performed across population subgroups stratified by gender, smoking status, drinking status, residence, marital status, and education status to investigate the predictive performance of the indices for stroke risk in different populations. Based on the ROC curves, the optimal cutoff value was determined, and the corresponding sensitivity and specificity were calculated to evaluate their core classification performance. The number needed to diagnose was also computed to assess screening efficiency. Finally, subgroup and interaction analyses were performed based on strata including age, gender, marital status, residence, education status, smoking status, drinking status, and asthmatic condition to examine the associations and variations of the study indices across different subgroups. To evaluate the robustness of the primary findings against potential unmeasured confounding, E-value analysis was performed. The E-value quantifies the minimum strength of association that an unmeasured confounder would need to have with both the exposure and the outcome simultaneously to fully explain away the observed association.

R statistical software (version 4.4.2) was employed for all analyses, and statistical significance was set at a two-tailed *p*-value < 0.05.

## 3 Results

### 3.1 Baseline characteristics of participants

A total of 907 participants (411 men and 496 women) were enrolled, of whom 70.78% lived in rural regions. The mean age was 59.46 years, with average BRI and CTI values of 4.16 ± 1.50 and 8.73 ± 0.86, respectively. During follow-up, 81 individuals (8.93%) experienced stroke onset, with an average age of 62.06 years. Compared to participants who remained stroke-free, those who developed stroke were generally older, more often unmarried, and tended to reside in urban areas. Baseline characteristics of participants according to stroke status are detailed in Table 1.

**Table 1:** Baseline characteristics by stroke status.

Variable	Total	MCI without stroke	MCI with stroke	*p*
	(*n* = 907)	(*n* = 826)	(*n* = 81)	
Sociodemographic characteristics				
Age	59.46 ± 9.43	59.20 ± 9.41	62.06 ± 9.28	0.009*
Gender				0.216
Woman	496 (54.69%)	457 (55.33%)	39 (48.15%)	
Man	411 (45.31%)	369 (44.67%)	42 (51.85%)	
Marital status				0.168
Non-married	113 (12.46%)	99 (11.99%)	14 (17.28%)	
Married	794 (87.54%)	727 (88.01%)	67 (82.72%)	
Residence				0.550
Urban	265 (29.22%)	239 (28.93%)	26 (32.10%)	
Rural	642 (70.78%)	587 (71.07%)	55 (67.90%)	
Education status				0.656
High school or above	17 (1.87%)	16 (1.94%)	1 (1.23%)	
High school or below	890 (98.13%)	810 (98.06%)	80 (98.77%)	
Lifestyle factors				
Smoking status				0.121
No	565 (62.29%)	521 (63.08%)	44 (54.32%)	
Yes	342 (37.71%)	305 (36.92%)	37 (45.68%)	
Drinking status				0.511
No	535 (58.99%)	490 (59.32%)	45 (55.56%)	
Yes	372 (41.01%)	336 (40.68%)	36 (44.44%)	
Clinical characteristics				
Asthma				0.895
No	854 (94.16%)	778 (94.19%)	76 (93.83%)	
Yes	53 (5.84%)	48 (5.81%)	5 (6.17%)	
BRI	4.16 ± 1.50	4.13 ± 1.48	4.40 ± 1.64	0.118
CTI	8.73 ± 0.86	8.71 ± 0.86	8.93 ± 0.87	0.028*

*p < 0.05, it indicates that the comparison is statistically significant.

### 3.2 Association of BRI and CTI with stroke risk in patients with MCI

**Figure 2:**
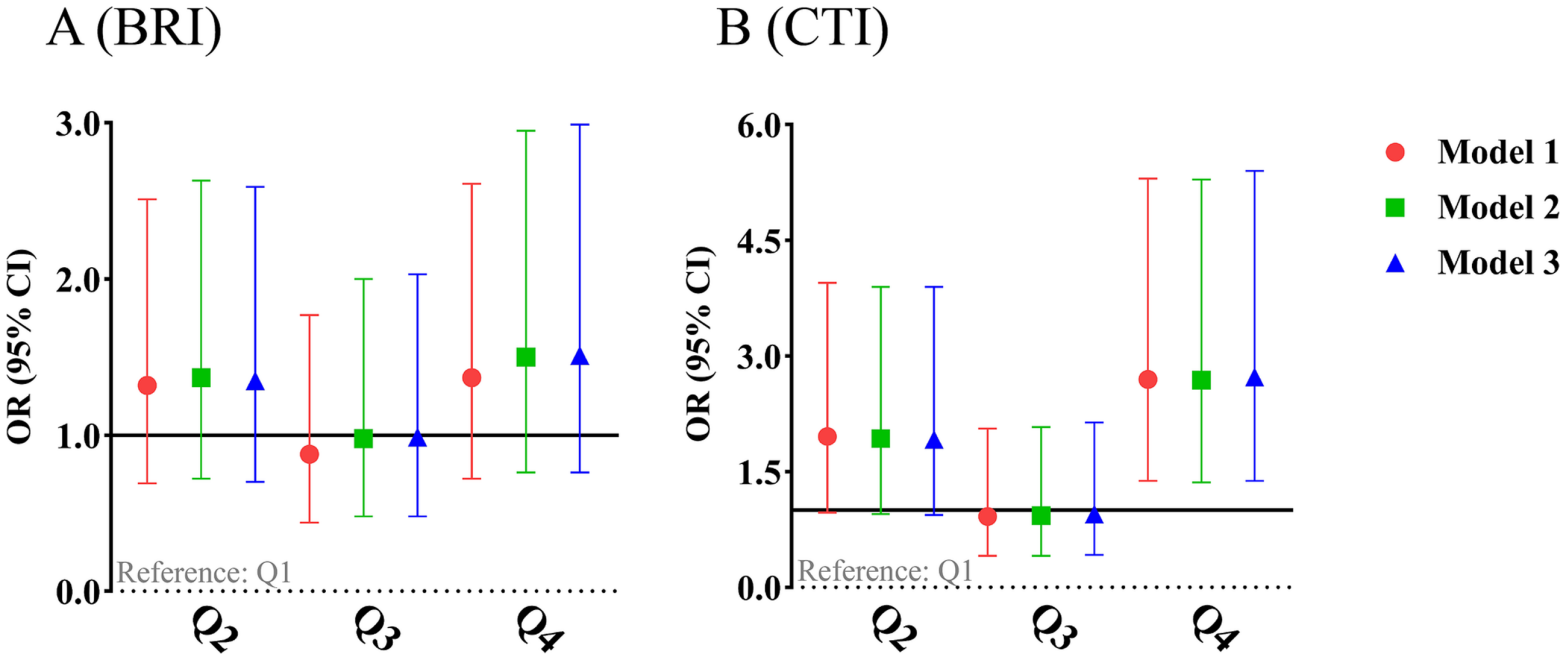
Forest plots for the association of BRI and CTI with stroke in MCI patients. **(A)** BRI; **(B)** CTI. Quartiles were defined based on the distribution of BRI and CTI levels; Q1 represents the lowest quartile (reference group), while Q2-Q4 represent higher quartiles compared with Q1, data are presented as odds ratios with 95% confidence intervals. (Model 1: unadjusted for any covariates; Model 2: adjusted for gender, age; Model 3: adjusted for gender, age, residence, marital status, education status, smoking status, drinking status, asthma).

Multivariable logistic regression analysis revealed no significant association between increased BRI and stroke occurrence in the MCI population compared to the lowest quartile (Q1) (OR: 1.12, 95% CI: 0.97 ~ 1.30, *p* > 0.05). Furthermore, no significant increase in stroke risk was observed for upper quartiles (Q2, Q3, Q4) when each was compared individually to Q1 (Figure 2A, Table 2). Additional trend tests showed no significant trend association between BRI levels and stroke risk in the MCI population after adjusting for potential confounding covariates (P for trend > 0.05), indicating no dose–response relationship (Table 2). However, after accounting for relevant covariates, multivariable logistic regression (Model 3) revealed a significant 34% elevated stroke risk per one-unit increment in CTI among individuals with MCI (OR: 1.34, 95% CI: 1.04–1.72, *p* < 0.05) (Table 2). Additionally, compared to individuals in the lowest quartile (Q1), those in the highest quartile (Q4) experienced a notably greater risk of stroke (OR: 2.70, 95% CI: 1.38–5.30, *p* < 0.005), a relationship that persisted after adjustments. By contrast, no significant risk increase was observed in the Q2 and Q3 groups (Figure 2B). Further trend tests revealed a significant increasing trend between CTI levels and stroke risk in the MCI population (P for trend < 0.05), indicating a clear dose–response relationship independent of potential confounders (Table 2). To assess the potential influence of unmeasured confounding on the primary association between CTI and stroke risk, E-value analysis was conducted. The E-value for the point estimate (OR: 1.34) was 2.01, and the E-value for the lower bound of the 95% confidence interval (OR: 1.04) was 1.24. These results indicate that an unmeasured confounder would need to be associated with both CTI and stroke by a risk ratio of at least 2.01-fold to fully nullify the observed association, and a confounder of at least 1.24-fold would be required to shift the confidence interval boundary to the null value.

**Table 2:** Associations of the BRI and CTI with stroke risk.

Variable	Model 1			Model 2			Model 3		
	OR	95%CI	*p*	OR	95%CI	*p*	OR	95%CI	*p*
BRI	1.12	(0.97, 1.30)	0.118	1.16	(1.00, 1.35)	0.058	1.17	(1.00, 1.36)	0.054
BRI quartile									
Q1	Ref			Ref			Ref		
Q2	1.32	(0.69, 2.51)	0.406	1.37	(0.72, 2.63)	0.340	1.35	(0.70, 2.59)	0.374
Q3	0.88	(0.44, 1.77)	0.722	0.98	(0.48, 2.00)	0.955	0.99	(0.48, 2.03)	0.981
Q4	1.37	(0.72, 2.61)	0.333	1.50	(0.76, 2.95)	0.238	1.51	(0.76, 2.99)	0.237
*P* for trend (BRI)	0.570			0.403			0.381		
CTI	1.32	(1.03, 1.69)	0.029*	1.33	(1.03, 1.71)	0.026*	1.34	(1.04, 1.72)	0.025*
CTI quartile									
Q1	Ref			Ref			Ref		
Q2	1.96	(0.97, 3.95)	0.061	1.93	(0.95, 3.90)	0.068	1.92	(0.94, 3.90)	0.072
Q3	0.92	(0.41, 2.06)	0.837	0.93	(0.41, 2.08)	0.854	0.95	(0.42, 2.14)	0.899
Q4	2.70	(1.38, 5.30)	0.004*	2.69	(1.36, 5.29)	0.004*	2.73	(1.38, 5.40)	0.004*
*P* for trend (CTI)	0.020*			0.021*			0.019*		

OR, Odds ratio; CI, Confidence interval;*p < 0.05.

Model 1: unadjusted for any covariates.

Model 2: adjusted for gender, age.

Model 3: adjusted for gender, age, marital status, residence,education status, smoking status, drinking status, asthma.

### 3.3 Impact of smoking and drinking status on CTI predictive performance

Both alcohol and tobacco use are recognized risk factors for increasing the risk of dementia, cognitive impairment, and stroke in middle-aged and older adults. Therefore, we conducted threshold effect analysis, ROC analysis, and best threshold analysis to evaluate the predictive value of CTI within these two key high-risk subgroups of MCI patients: smokers and alcohol drinkers.

#### 3.3.1 Stratification by smoking status

**Figure 3:**
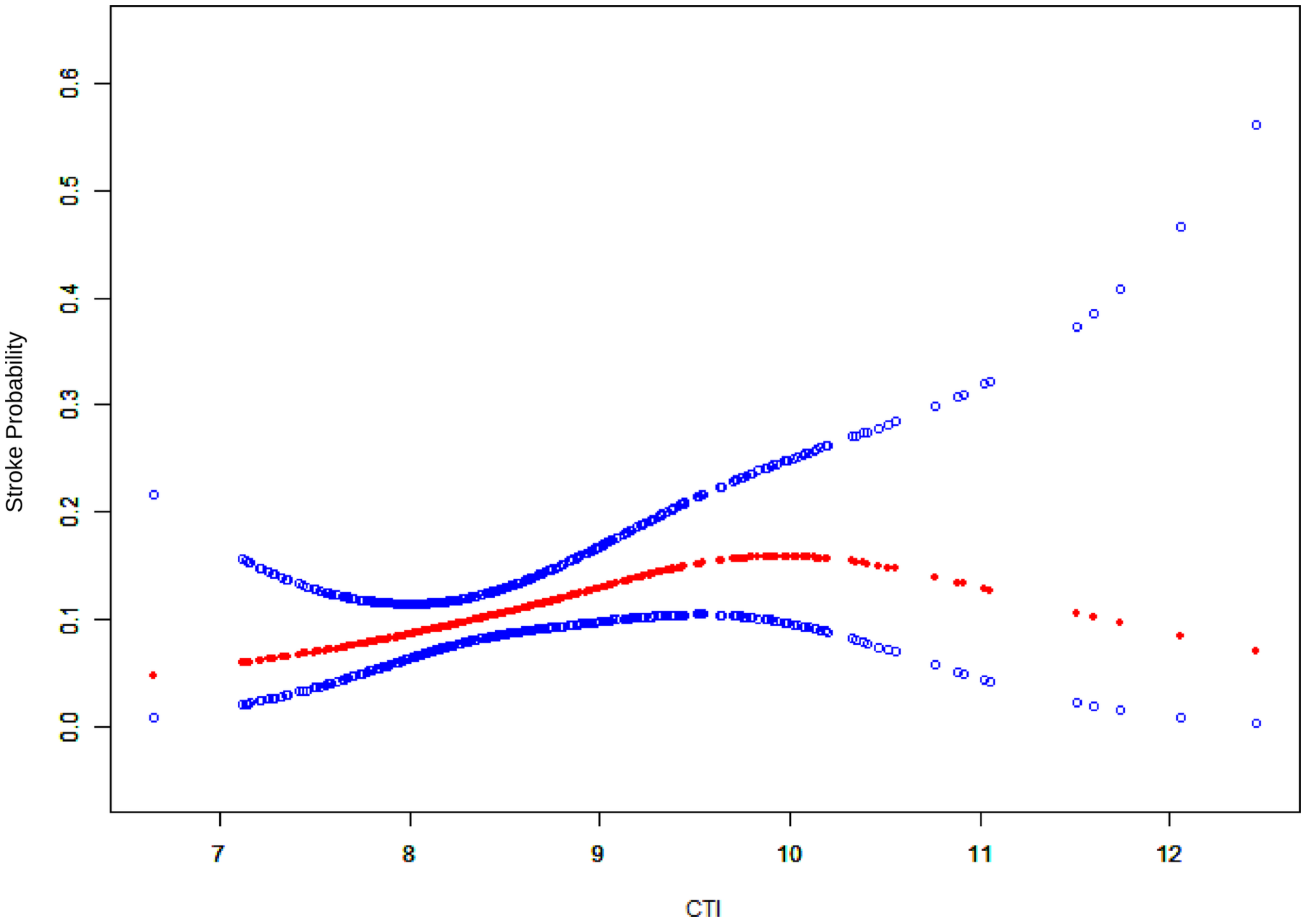
Dose–response relationship between CTI and stroke risk stratified by smoking status, fitted using a generalized additive model (GAM). The red line represents the smoothing curve, and the blue shaded area indicates the 95% confidence interval. The dashed vertical line denotes the inflection point (CTI = 9.91) identified by piecewise linear regression. Red dots represent smokers; blue circles represent non-smokers.

As shown in Figure 3 and Table 3, stratification by smoking habits uncovered a nonlinear relationship between CTI and stroke risk specifically among smokers with MCI (log-likelihood ratio test, *p* < 0.05). When CTI was lower than 9.91, every additional unit increase corresponded to a significant 81% rise in stroke risk (OR: 1.81, 95% CI: 1.09–3.01, p < 0.05). Conversely, no significant CTI-stroke association was detected at CTI values exceeding 9.91 (OR: 0.21, 95% CI: 0.02–2.62, *p* > 0.05). Additionally, differences in effect on either side of the inflection point were not statistically significant. In contrast, among non-smokers with MCI, no significant threshold effect was detected (log-likelihood ratio test, p > 0.05).

**Table 3:** Threshold effect analysis of CTI on stroke risk stratified by smoking status.

Effect parameter	Non-smoker			Smoker		
	OR	95%CI	*p*	OR	95%CI	*p*
Model 1						
Linear effect	1.37	(0.97, 1.94)	0.071	1.27	(0.89, 1.82)	0.184
Model 2						
Inflection point (K)	7.64			9.91		
Effect size (segment < K)	0.16	(0.01, 2.14)	0.167	1.81	(1.09, 3.01)	0.022*
Effect size (segment > K)	1.51	(1.05, 2.16)	0.025*	0.21	(0.02, 2.62)	0.225
Log-likelihood ratio test	0.156			0.036*		

OR, Odds ratio; CI, Confidence interval; *p < 0.05.

Model 1: Linear regression model without a threshold.

Model 2: Piecewise linear regression model.

**Figure 4:**
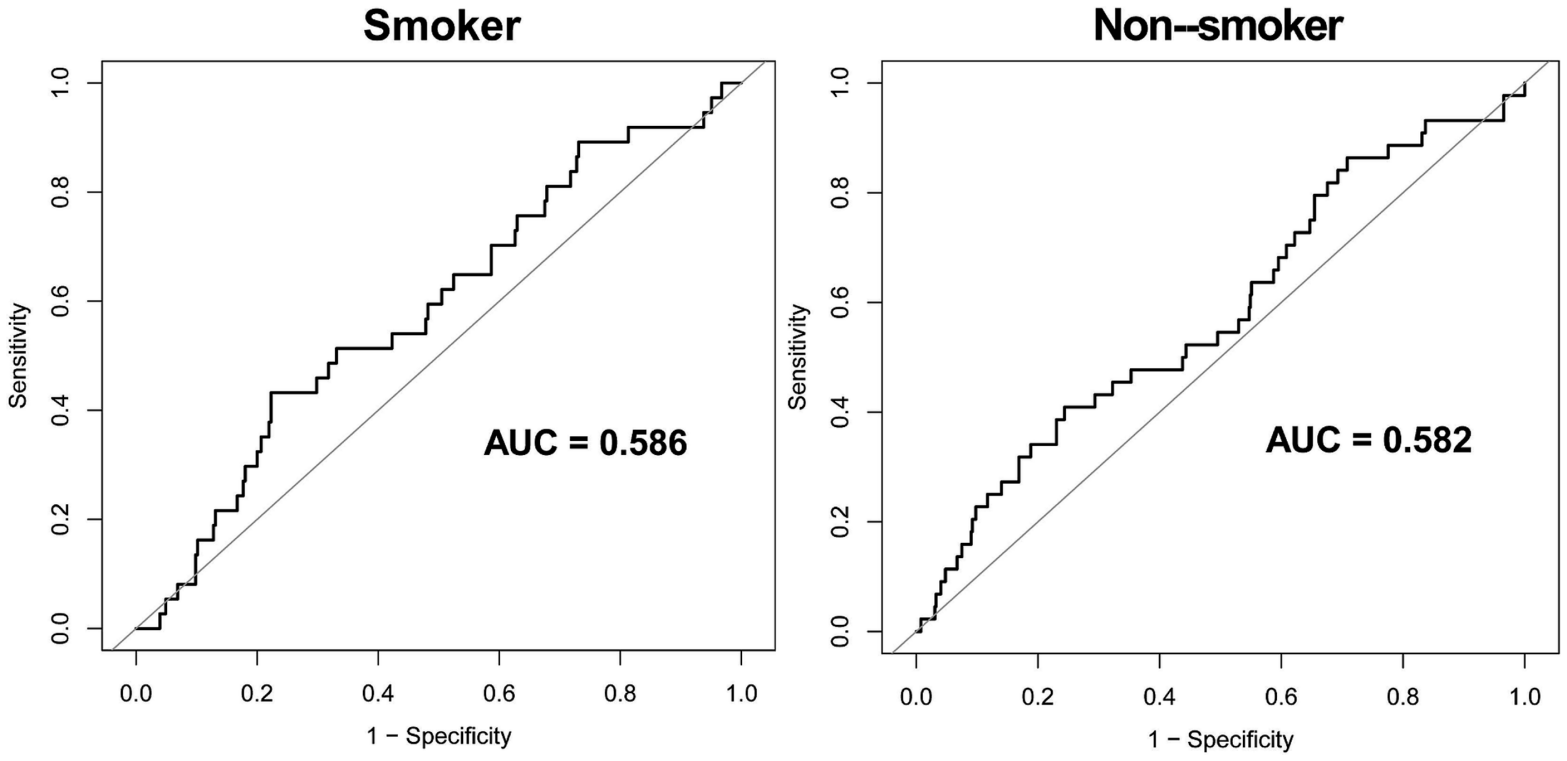
ROC Curves by smoking status (*X*-axis = 1-specificity; False Positive Rate, FPR); (*Y*-axis = sensitivity; True Positive Rate, TPR). The curve plots sensitivity against 1-specificity across various cutoff points, the diagonal line represents the reference line of no discrimination, curve indicates model performance.

However, despite the differences in threshold effect patterns between the two groups, ROC analysis results indicated that CTI had comparable predictive ability in both, with an Area Under the Curve (AUC) of 0.586 for smokers and 0.582 for non-smokers (Figure 4). Furthermore, optimal threshold analysis revealed nearly identical optimal risk cutoff values for both groups (smoker = 9.22, non-smoker = 9.24) (Supplementary Table S1).

#### 3.3.2 Stratification by drinking status

**Figure 5:**
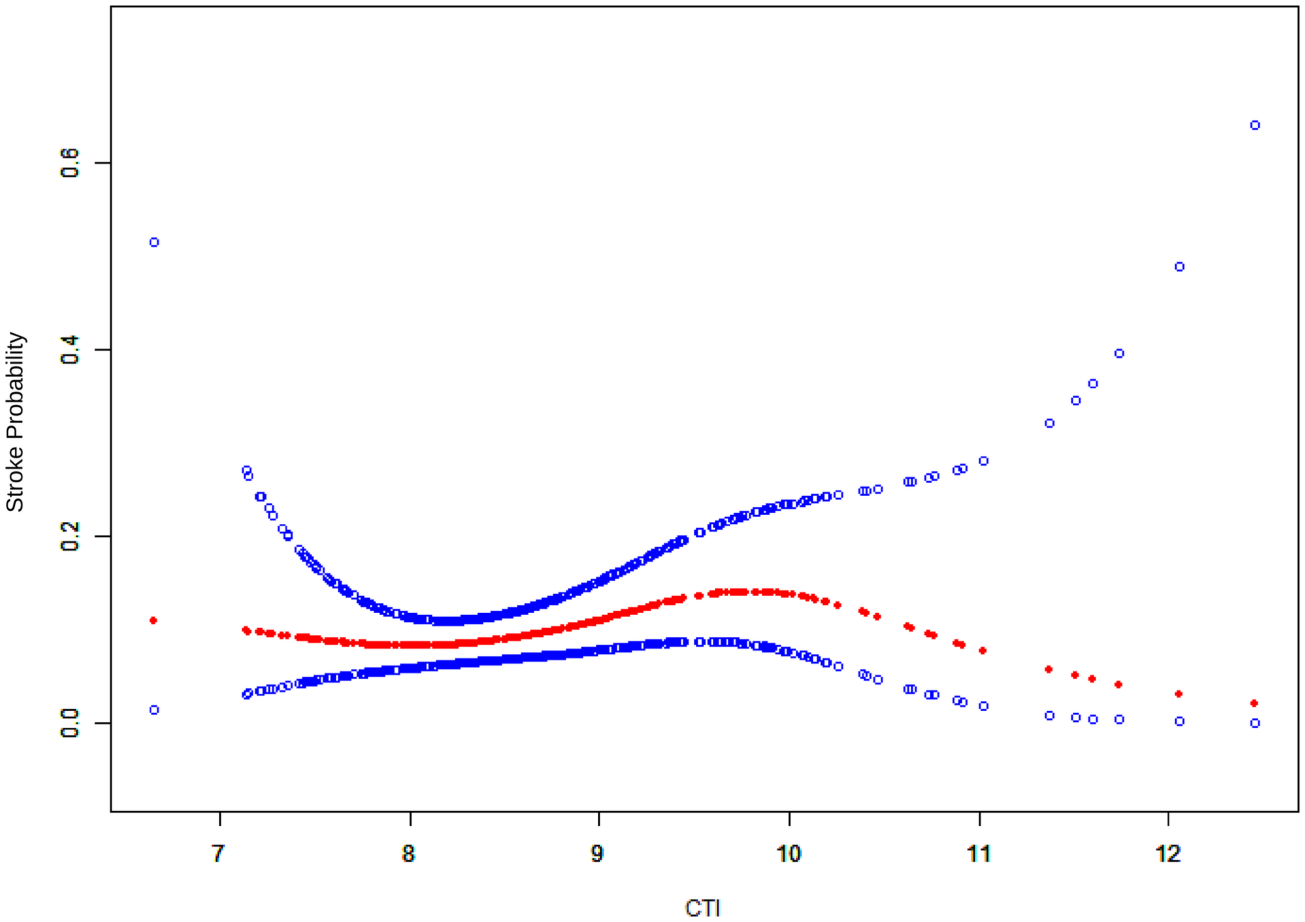
Dose–response relationship between CTI and stroke risk stratified by drinking status, fitted using a generalized additive model (GAM). The red line represents the smoothing curve, and the blue shaded area indicates the 95% confidence interval. Red dots represent drinkers; blue circles represent non-drinkers.

Threshold effect analysis of CTI stratified by drinking status showed no significant threshold effects among either drinkers or non-drinkers with MCI (log-likelihood ratio test, *p* > 0.05). These findings suggest a linear or no association between CTI and stroke risk in these groups. Detailed results are presented in Figure 5 and Table 4.

**Table 4:** Threshold effect analysis of CTI on stroke risk stratified by drinking status.

Effect parameter	Non-drinker			Drinker		
	OR	95%CI	*P*	OR	95%CI	*p*
Model 1						
Linear effect	1.52	(1.08, 2.13)	0.015*	1.13	(0.77, 1.64)	0.540
Model 2						
Inflection point (K)	7.56			9.91		
Effect size (segment < K)	0.22	(0.01, 5.35)	0.349	1.52	(0.91, 2.54)	0.110
Effect size (segment > K)	1.61	(1.13, 2.29)	0.008*	0.16	(0.01, 3.75)	0.254
Log-likelihood ratio test	0.293			0.065		

OR, Odds ratio; CI, Confidence interval;*p < 0.05.

Model 1: Linear regression model without a threshold.

Model 2: Piecewise linear regression model.

**Figure 6:**
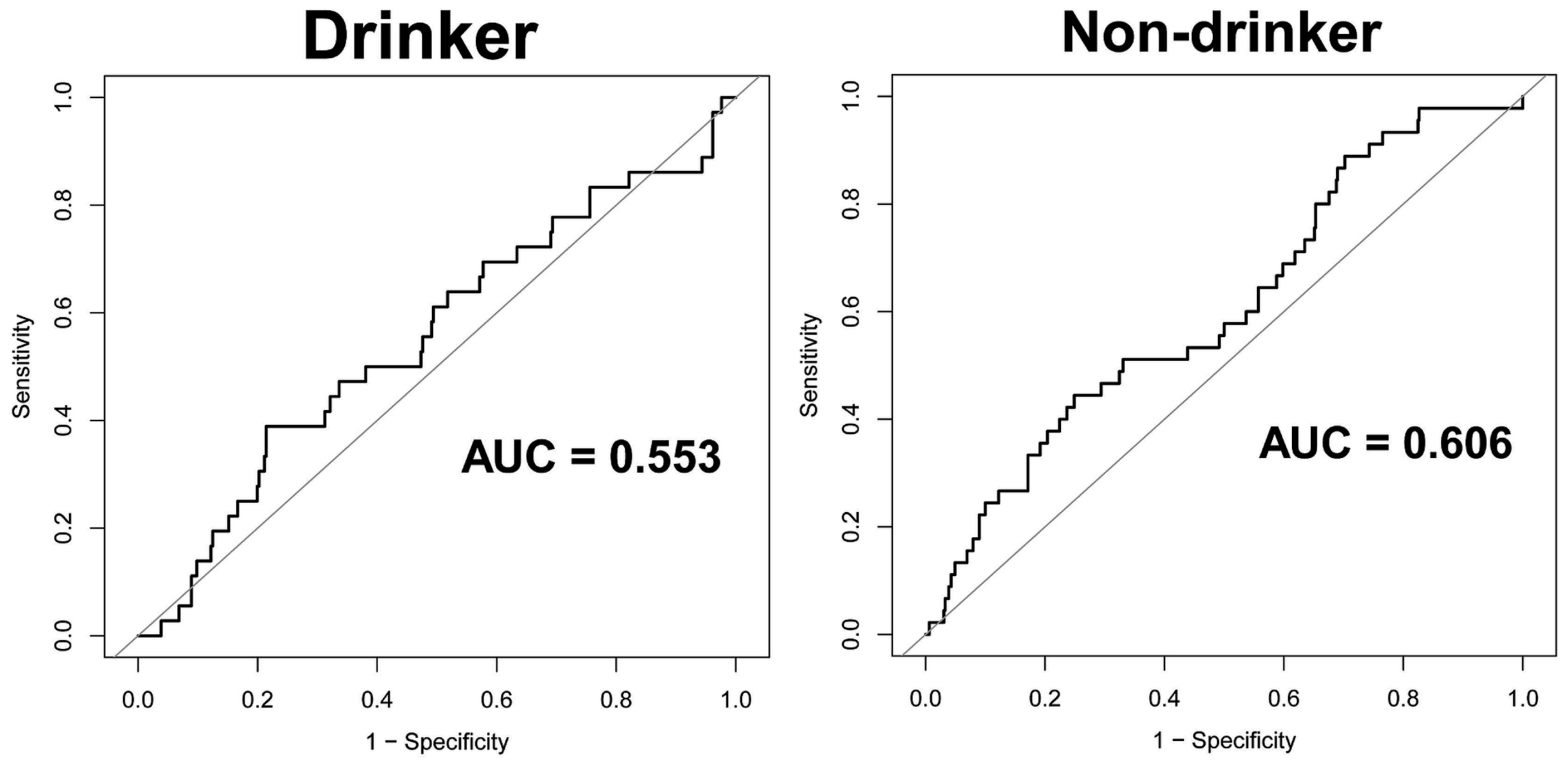
ROC Curves by drinking status (*X*-axis = 1-specificity; False Positive Rate, FPR); (*Y*-axis = sensitivity; True Positive Rate, TPR). The curve plots sensitivity against 1-specificity across various cutoff points, the diagonal line represents the reference line of no discrimination, curve indicates model performance.

Correspondingly, we further evaluated the predictive performance of CTI in these two groups. The AUC was 0.553 for drinkers and 0.606 for non-drinkers (Figure 6). While CTI demonstrated modest predictive ability in both groups, their optimal risk cutoff values were also relatively close (drinker = 9.21, non-drinker = 9.28) (Supplementary Table S1).

### 3.4 Stratified analysis of CTI predictive performance by other key factors

To assess the distribution of the CTI predictive value in diverse MCI populations, stratified ROC and optimal threshold analyses were extended to key demographic factors, complementing the earlier focus on smoking and alcohol use. The analyses revealed that CTI demonstrated a certain level of predictive ability across all subgroups, with relatively concentrated AUC values (0.556 for males, 0.614 for females; 0.593 for married, 0.527 for unmarried; 0.590 for urban residents, 0.577 for rural residents; 0.581 for lower education group) (Supplementary Figures S1–S4). Although CTI demonstrated consistent predictive performance across subgroups, the AUC values ranging from 0.55 to 0.61 indicated low-to-moderate discriminatory ability, suggesting that CTI alone is insufficient as a standalone screening tool for stroke risk in clinical practice. Its value lies primarily in serving as a complementary indicator to augment existing clinical risk assessment frameworks, and its independent clinical utility warrants confirmation in future prospective studies. Notably, the higher education subgroup was excluded from formal analysis due to an extremely small sample size (*n* = 17) and only one incident stroke, which precludes reliable ROC curve fitting and cutoff determination.

Based on the ROC analyses, the optimal CTI cutoff values across subgroups ranged from 8.20 to 9.28(See Supplementary Table S1 for details), which may serve as preliminary clinical reference thresholds for stroke risk stratification in MCI patients. Enhanced cerebrovascular monitoring is recommended for MCI patients whose CTI exceeds these thresholds. Regarding intervention targets, CTI integrates triglycerides, fasting plasma glucose, and CRP, suggesting that comprehensive interventions targeting dyslipidemia, insulin resistance, and chronic inflammation may represent potential therapeutic directions.

### 3.5 Subgroup and interaction analyses

**Figure 7:**
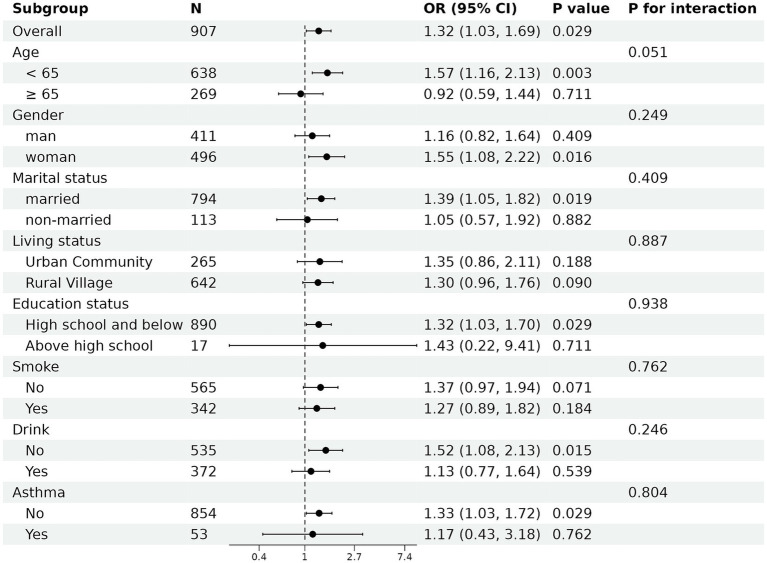
Forest plot of subgroup analysis of the association between CTI and stroke risk in the MCI population.

To further explore the association between CTI and stroke risk in MCI patients, subgroup and interaction analyses were conducted based on additional stratifications. (Figure 7). Nearly all subgroups displayed increased stroke incidence alongside rising CTI. Significant relationships emerged in subgroups characterized by age under 65 (OR: 1.57, 95% CI: 1.16–2.13), females (OR: 1.55, 95% CI: 1.08–2.22), married individuals (OR: 1.39, 95% CI: 1.05–1.82), higher educational levels (OR: 1.32, 95% CI: 1.03–1.70), non-drinkers (OR: 1.52, 95% CI: 1.08–2.13), and absence of asthma history (OR: 1.33, 95% CI: 1.03–1.72) (*p* < 0.05). Interaction analysis showed no statistically significant interaction between age and the CTI-stroke relationship (P for interactio*n* = 0.051), suggesting that age does not significantly modify this association. No notable interactions were found regarding sex, marital status, residence, education, tobacco use, alcohol intake, or asthma history.

## 4 Discussion

Based on a secondary analysis of a national prospective cohort, this study establishes, for the first time, a significant relationship between CTI, a biomarker integrating metabolic and inflammatory pathways, and stroke risk in individuals with MCI. In contrast, no substantial relationship emerged between BRI levels and stroke incidence. These results lend crucial epidemiological support to the proposed interactive impairment mechanism involving metabolism, cognitive function, and cerebrovascular integrity.

CTI represents an innovative index derived from integrating CRP with TyG (20). CRP is frequently utilized clinically as an indicator of systemic inflammation, whereas TyG serves as a straightforward proxy for insulin resistance evaluation. Although the hyperinsulinemic-euglycemic clamp is the recognized gold standard, TyG is widely preferred in clinical settings due to its minimally invasive nature, affordability, ease of use, and notable accuracy (24, 25). Earlier research has firmly linked insulin resistance to stroke (26), proposing systemic inflammation as a potential mediator (27). Originally conceived to predict cancer outcomes, accumulating evidence now supports the association between elevated CTI and stroke risk (28, 29). As for the BRI, which primarily reflects abdominal obesity, its relationship with stroke risk demonstrates a nonlinear positive correlation (30).

The prevalence of modern high-sugar diets has led to an increasing incidence of metabolic diseases, which are recognized risk factors for stroke. The pathological mechanisms underlying these diseases involve the activation of pathways such as Nuclear Factor kappa-light-chain-enhancer of activated B cells (NF-κB) and Tumor Necrosis Factor-alpha (TNF-*α*), which induce insulin resistance and chronic systemic inflammation (31–33). Systemic inflammation can compromise the integrity of the blood–brain barrier, enabling inflammatory substances to enter the central nervous system. This infiltration process can subsequently damage neuronal and glial structures, contributing to neurological disorders such as stroke and Alzheimer’s disease (34). The Fibroblast Growth Factor 23 (FGF23)-Klotho axis, a key endocrine regulator of insulin resistance and fatty acid metabolism, can elevate blood phosphorus levels when dysfunctional, causing vascular smooth muscle glycosylation abnormalities and promoting *de novo* cholesterol synthesis, thereby increasing atherosclerosis and stroke risk (35–37). Similarly, metabolic disorders are significant risk factors for MCI, contributing to varying degrees of cognitive dysfunction (38). Firstly, hyperglycemia and insulin resistance in type 2 diabetes activate oxidative stress and inflammatory pathways, accumulate advanced glycation end products, and reduce brain energy utilization efficiency, collectively exacerbating neuronal damage and synaptic dysfunction (39, 40). Secondly, obesity affects neuroglial cell activation through circulating saturated fatty acids, and its comorbidity with type 2 diabetes accelerates neuroinflammation and cognitive decline in Alzheimer’s disease (41).

In summary, MCI and stroke undoubtedly share a close bidirectional relationship, with systemic metabolic disorders centered on insulin resistance likely forming their common pathophysiological basis. This “metabolism-cognition-cerebrovascular” interactive impairment framework provides the theoretical foundation for the present study. Based on this framework, we introduced two composite indices, CTI and BRI, to quantitatively assess key risk components from metabolic inflammation and body shape distribution dimensions, respectively. Our data demonstrate a robust positive correlation between CTI levels and stroke incidence within the MCI cohort. After comprehensive adjustments for demographic attributes, lifestyle behaviors, and additional confounding influences, a single-unit elevation in CTI corresponded, on average, to a 34% heightened stroke risk. Although statistically significant risk enhancements were largely confined to participants exhibiting the highest CTI values, the observed relationship displayed a distinct dose–response trend independent of conventional confounders. These findings propose the utility of CTI as a potential predictive marker for identifying stroke-susceptible MCI patients. In contrast, BRI, reflecting central obesity, did not demonstrate independent predictive value in the MCI population. This finding may be attributed to two key biological limitations of BRI in this specific context. First, MCI patients frequently exhibit sarcopenia and age-related weight loss, which can reduce the representativeness of waist circumference-derived indices as proxies for metabolic risk. Recent evidence further suggests that composite metabolic-body composition indices outperform BRI alone in this population (42). Second, stroke risk in MCI patients appears to be predominantly driven by neuroinflammation, dyslipidemia, and insulin resistance rather than body fat distribution per se (38, 43), which are the metabolic dimensions captured by CTI rather than BRI. These considerations suggest that BRI, as a body shape index, may not adequately capture the pathophysiological mechanisms most relevant to stroke risk in MCI patients.

To enable more precise risk assessment, we performed systematic stratified analyses considering that certain behavioral factors and characteristics might introduce heterogeneity in the predictive value of the CTI. The ROC results indicated that CTI, as a simple screening tool, exhibited relatively stable predictive performance for stroke across different populations. Subgroup analysis suggested that particular attention should be paid to married female patients with MCI under 65 years of age, as this group faces a higher future risk of stroke. Extensive previous research has confirmed that smoking and alcohol consumption elevate the risk of cardiovascular and neurodegenerative diseases. Tobacco use leads to disrupted functional connectivity within resting-state networks (44), thereby reducing functional compensation in MCI patients, while smoking independently increases stroke risk—a risk that remains elevated even when switching from traditional to electronic cigarettes (45). Although low-level alcohol consumption is considered potentially cardioprotective, heavy drinking significantly raises the risk of stroke (46) and correspondingly increases the prevalence of MCI (47). Given this, our analysis specifically focused on these two high-risk behaviors. The results revealed a nonlinear association between CTI and stroke risk among MCI patients who smoke, characterized by a significant association at lower CTI levels (< 9.91) and no statistically significant association above this threshold (> 9.91). This phenomenon may be explained by a saturation effect: smoking is a strong pro-inflammatory and pro-atherogenic risk factor that markedly elevates baseline cerebrovascular risk via endothelial dysfunction, oxidative stress, and systemic inflammation (48, 49). Accordingly, further increases in CTI beyond 9.91 may only lead to a marginal rise in stroke risk, resulting in a plateau of the dose–response relationship at high CTI levels. On the other hand, several unmeasured confounding factors may account for the non-significant association between CTI and stroke risk among smokers with CTI > 9.91. These factors, which merit further investigation in future research, include the following: first, smoking intensity and cumulative exposure expressed as pack-years, as our study only distinguished smoking status dichotomously without quantifying dose; second, smoking cessation status and duration, which can substantially alter metabolic phenotype and vascular risk profile; third, concomitant use of statins or antiplatelet agents, which may modify the dose–response relationship between CTI and stroke risk; and fourth, inflammatory biomarkers such as high-sensitivity CRP and oxidative stress indices, which may mediate the interaction between smoking and elevated CTI. These findings are derived from an exploratory analysis, and larger sample sizes are recommended in future studies to further investigate this threshold effect. However, no clear threshold effect was observed based on drinking status. The complex relationship between alcohol consumption and stroke risk may explain this finding. Variations in stroke risk are closely related to the amount and type of alcohol consumed, with certain drinking patterns potentially reducing risk (46). Our study categorized participants only by drinking status, which may have obscured more nuanced nonlinear relationships. Future research should collect more detailed information on alcohol intake to explore potential distinct association patterns.

## 5 Strengths and limitations

Strengths: This prospective cohort study establishing the CTI as an independent predictor of stroke in MCI patients provides crucial human evidence for the “metabolism-cognition-cerebrovascular” interaction hypothesis. The large, nationally representative CHARLS sample enhances credibility. Systematic stratified analyses confirmed result stability across populations, simultaneously aiding in identifying high-risk subgroups. CTI’s low cost facilitates widespread clinical implementation.

Limitations: Stroke outcomes based on self-reported physician diagnoses may involve recall bias and lack subtype specification. Generalizability is limited to Chinese older adults, and residual confounding remains possible. Additionally, the AUC values for CTI across all subgroups ranged from 0.55 to 0.61, reflecting low-to-moderate predictive performance, which limits its standalone clinical utility for stroke risk screening.

## 6 Conclusion

Among Chinese middle-aged and elderly MCI patients, the CTI, which reflects metabolic-inflammatory status, independently predicts stroke risk, with preliminary reference thresholds ranging from 8.20 to 9.28 across subgroups. Thus, prioritizing intrinsic metabolic dysfunction over body morphology enables precise risk stratification and guides future research on preventive pathway modulation.

## Data Availability

The original contributions presented in the study are included in the article/Supplementary material, further inquiries can be directed to the corresponding author.
