# Supplementary material for: Multifactorial stratified analysis of two novel metabolism-related indices for predicting stroke in mild cognitive impairment
Source: Front Neurol. 2026 Jul 13;17:1814327. doi: 10.3389/fneur.2026.1814327 (PMC13402179; doi:10.3389/fneur.2026.1814327)
Supplement: Supplementary file 1 [file Table_1.docx]

Supplementary Material

**Table S1.** Stratified best threshold analysis

| **Basis for stratification** | **Best threshold** | **Sensitivity** | **Specificity** | **Number for diagnose** |
| --- | --- | --- | --- | --- |
| Smoking status |  |  |  |  |
| smoker | 9.22 | 0.43 | 0.78 | 4.77 |
| non-smoker | 9.24 | 0.41 | 0.76 | 6.05 |
| Drinking status |  |  |  |  |
| drinker | 9.21 | 0.39 | 0.79 | 5.73 |
| non-dinker | 9.28 | 0.44 | 0.75 | 5.12 |
| Gender |  |  |  |  |
| man | 9.14 | 0.38 | 0.78 | 6.19 |
| woman | 8.27 | 0.92 | 0.30 | 4.40 |
| Marital status |  |  |  |  |
| married | 9.22 | 0.45 | 0.77 | 4.64 |
| non-married | 8.27 | 0.79 | 0.34 | 7.74 |
| Living status |  |  |  |  |
| urban community | 9.23 | 0.54 | 0.71 | 4.00 |
| rural village | 8.20 | 0.85 | 0.32 | 5.61 |
| Education status |  |  |  |  |
| high school and below | 9.25 | 0.41 | 0.76 | 5.66 |

**
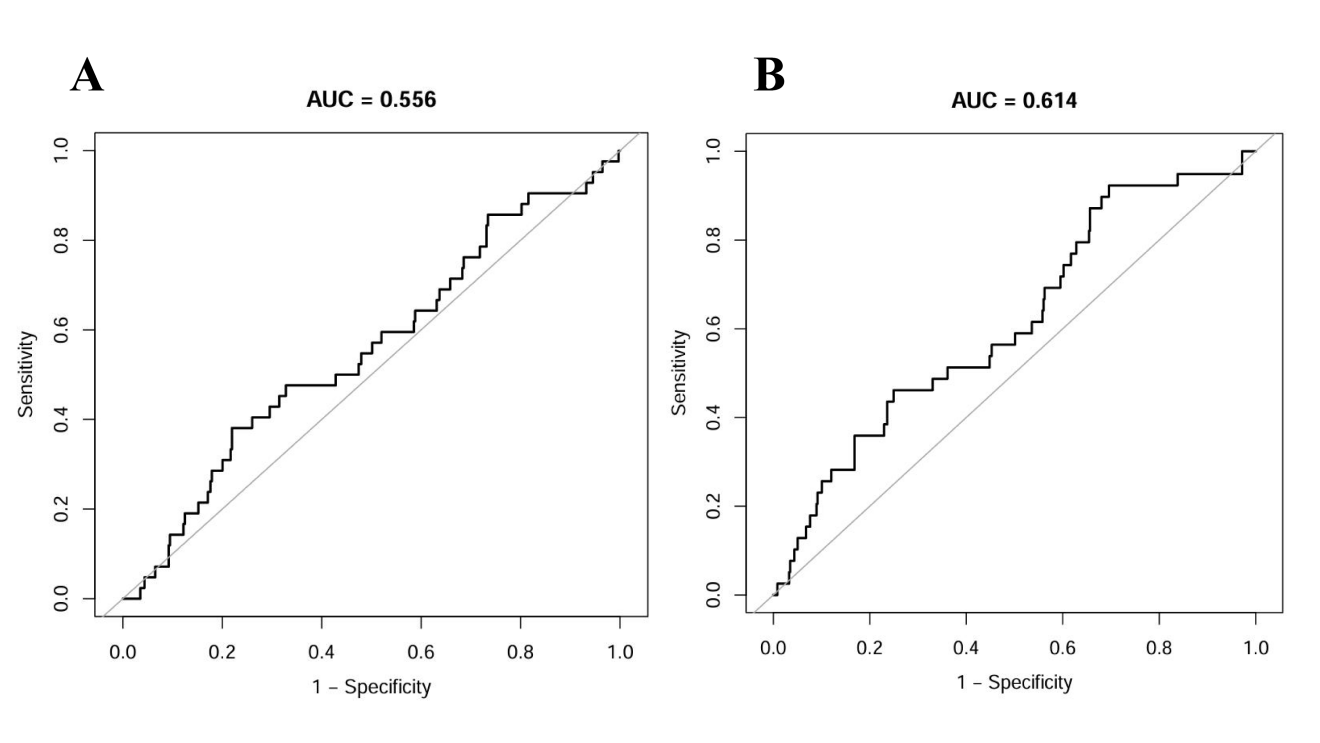
**

**Figure S1.** ROC Curves by Gender. **(A)** Man; **(B)** Woman.

**Figure S2.** ROC Curves by Marital Status. **(A)** Married; **(B)** Non-married.


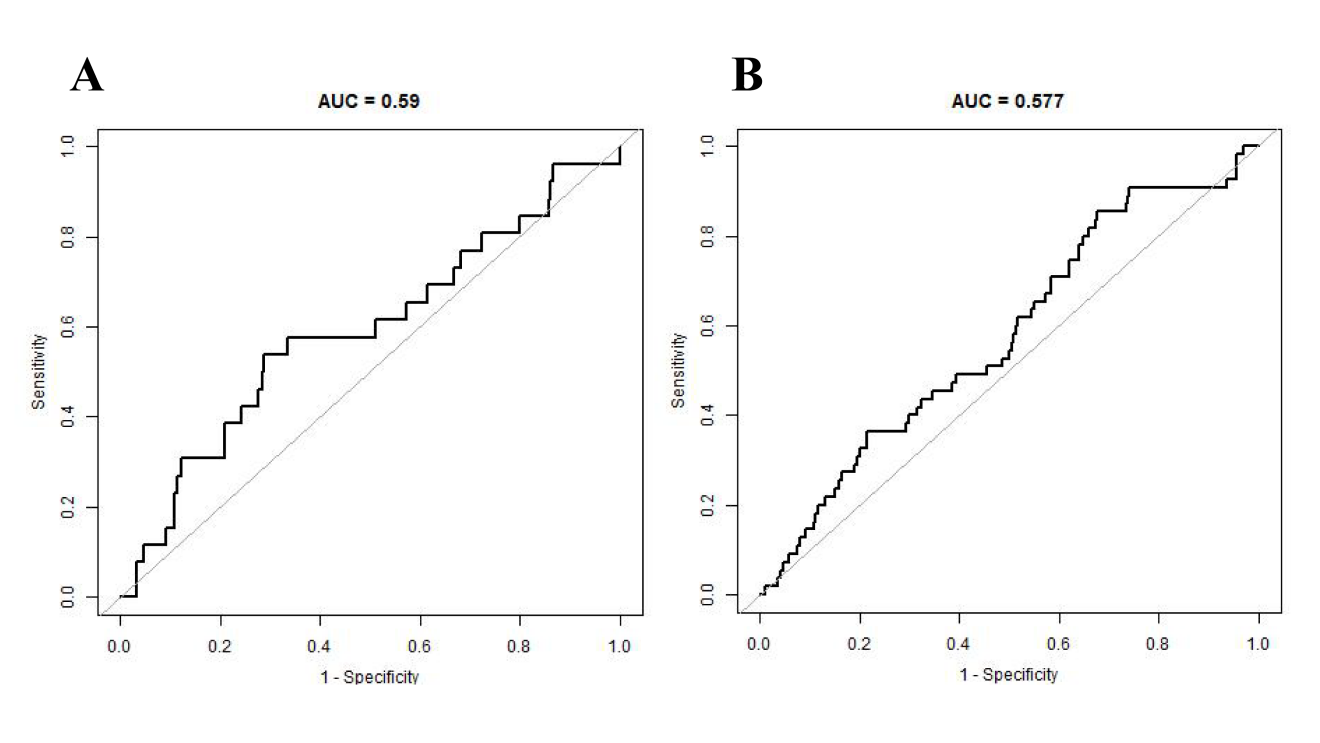


**Figure S3.** ROC Curves by Living Status. **(A)** Urban community; **(B)** Rural village.


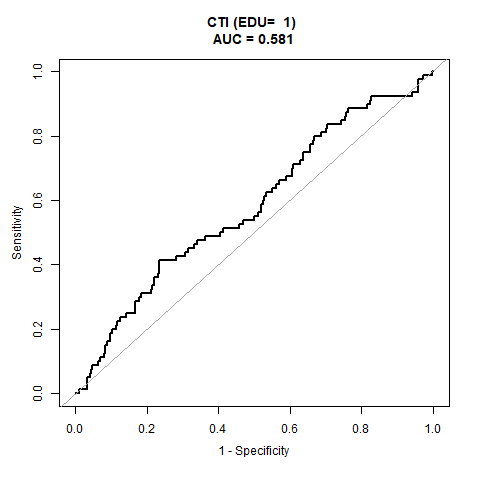


**Figure S4.** ROC Curves by Education Status (High School and Below) .
